# An observational prospective study of topical acidified nitrite for killing methicillin-resistant *Staphylococcus aureus *(MRSA) in contaminated wounds

**DOI:** 10.1186/1756-0500-4-458

**Published:** 2011-10-27

**Authors:** Anthony D Ormerod, Amjad AJ Shah, Hong Li, Nigel B Benjamin, Gail P Ferguson, Carlo Leifert

**Affiliations:** 1 Division of Applied Medicine, University of Aberdeen, Polwarth Building, Foresterhill, Aberdeen, AB24 2ZD, UK; 2 Royal Lancaster Infirmary, Ashton Road, Lancaster, LA1 4RP, UK; 3 Centre For Ecology & Hydrology, Lancaster Environment Centre, Bailrigg, Lancaster, LA1 4AP, UK; 4 Acute Medical Unit, Level 9, Derriford Hospital, Plymouth, PL6 8DH, UK; 5 Newcastle University, Nafferton Farm, Stocksfield, Northumberland, NE43 7XD, UK

## Abstract

**Background:**

Endogenous nitric oxide (NO) kills bacteria and other organisms as part of the innate immune response. When nitrite is exposed to low pH, NO is generated and has been used as an NO delivery system to treat skin infections. We demonstrated eradication of MRSA carriage from wounds using a topical formulation of citric acid (4.5%) and sodium nitrite (3%) creams co-applied for 5 days to 15 wounds in an observational prospective pilot study of 8 patients.

**Findings:**

Following treatment with topical citric acid and sodium nitrite, 9 of 15 wounds (60%) and 3 of 8 patients (37%) were cleared of infection. MRSA isolates from these patients were all sensitive to acidified nitrite *in vitro *compared to methicillin-sensitive *S. aureus *and a reference strain of MRSA.

**Conclusions:**

Nitric oxide and acidified nitrite offer a novel therapy for control of MRSA in wounds. Wounds that were not cleared of infection may have been re-contaminated or the bioavailability of acidified nitrite impaired by local factors in the tissue.

## Background

The widespread clinical use of antibiotics over the last 50 years has led to the emergence of resistant strains [1]. Methicillin-resistant *Staphylococcus aureus *(MRSA) was first noted in Great Britain in the early 60 s [2]. MRSA is a major cause of infections in humans worldwide, in both the community and the hospital [3]. Following targeted action, the incidence of MRSA bacteraemia has been falling since 2006 in the UK [3]. However, it still remains a considerable problem throughout Europe [4]. Surgical wounds are frequently colonised or significantly infected with MRSA [5]. MRSA is a major cause of surgical site infection which can delay hospital discharge [6]. In another study 23% of diabetic foot ulcers were infected [7]. MRSA infections are also a frequent cause of abscesses [8] and novel or better bactericidal agents that can be applied to wounds for decolonisation or prevention are urgently needed [9].

Acidified nitrite was devised as a novel means of liberating the bactericidal gas nitric oxide (NO) on the skin as a topical antibiotic therapy [10-12]. Briefly nitrite and hydrogen ions form nitrous acid (1) which is converted to dinitrogen trioxide (2), which dissociates into nitric oxide and nitrous oxide (3).

(1) $$\text{N}{\text{O}}_{{\text{2}}^{-}}+{\text{H}}^{+}⇄\text{HN}{\text{O}}_{\text{2}}$$

(2) $$2\text{HN}{\text{O}}_{\text{2}}⇄{\text{H}}_{2}\text{O + }{\text{N}}_{\text{2}}{\text{O}}_{\text{3}}$$

(3) $${\text{N}}_{2}{\text{O}}_{3}⇄\text{NO}+\text{N}{\text{O}}_{2}$$

We found that *Trichophyton mentagrophytes, T. rubrum, Candida albicans, Streptococcus pyogenes, S. aureus *and *Propionibacterium acnes *are all sensitive to acidified nitrite [12], with *S. aureus *being particularly sensitive. In the clinic, the concept of combining topical treatment with a nitrite containing cream and an acidic cream as a means of topical NO therapy has been proven [11,10] and used to treat *Mycobacterium ulcerans *causing Buruli ulcer [13] by co-application of creams containing 6% sodium nitrite and 9% citric acid.

As topical NO combining 3.0% (w/v) sodium nitrite and 4.5% (w/v) citric acid also facilitates experimental wound healing [14] it is an excellent candidate for decontaminating infected wounds. We hypothesised that acidified nitrite would be a useful agent in eliminating MRSA infection from the skin and aimed to demonstrate its ability to inhibit and kill MRSA in vitro and in a clinical plot study.

## Methods

### Subjects

We recruited hospitalised patients with a positive MRSA wound culture. Pregnant and lactating females and those with carriage of MRSA cultured from nose, axilla, groins, throat or sputum were excluded from the study to avoid recontamination of the wound. Patients taking systemic antibiotics or demonstrating additional pathogens in the wound swabs were also excluded.

Initial swabs of the nose, throat, axillae, perineum and any unhealed wounds were taken to assess eligibility for the study. These were all repeated in a routine fashion by the nursing staff in accordance with well defined infection control protocols at baseline, day five of treatment and two and four days after stopping topical acidified nitrite therapy to assess recurrence of infection. Those who developed nasal carriage during the study were treated with nasal applications of mupirocin.

### Intervention

Treatment was applied to the infected wound twice daily for 5 days. Trained nursing staff co-applied equal amounts of 4.5% citric acid in aqueous cream and 3% sodium nitrite in aqueous cream mixed directly on the infected wound and surrounding skin which was then covered with a light gauze dressing. Once daily dressings were changed using aseptic technique, wounds were irrigated with sterile saline and the cream and a sterile dressing reapplied.

### Safety

Acidified nitrite has been used safely in previous studies [11,13,15,16]. Citric acid and sodium nitrite were chosen as having optimum stability and safety for use in humans, citric acid being a naturally occurring agent in the diet and an approved ingredient in cosmetics and skin care products. Sodium nitrite also occurs naturally in the diet in vegetables and meat and is a permitted food preservative, but systemic doses of 4.5 g are sufficient to kill a human through methaemoglobinaemia. There have also been concerns about systemic formation of N-nitrosothiols which may have carcinogenic potential in the gut [17]. The concentrations of sodium nitrite and citric acid used represent a 2:1 molar ratio of sodium nitrite to citric acid to ensure sufficient acid is available to react with the nitrite to prevent residual nitrite being systemically absorbed leading to methaemoglobinaemia. Citric acid and sodium nitrite have been used safely for topical application to the skin up to 9 and 13.5% twice daily for up to 4 months in unpublished studies (Pro-Strakan data on file) and on ulcerated skin has been used safely in humans up to 9% and 6% respectively in a published study [18], where positive effects on wound healing were observed. Two year carcinogenicity studies have been performed on 330 mice without significant oncogenesis. In pilot studies and Phase 1&II studies in man over 700 subjects have received skin treatment with concentrations of acidified nitrite of up to 9% sodium nitrite and 13.5% citric acid for up to 3-4 months with no treatment related serious adverse events and only mild skin irritation and staining occurring as side effects (Pro-Strakan data on file).

### MRSA Screening

All clinical samples were processed by standard culture methods on horse blood agar and MacConkey agar. Suspect colonies were confirmed as *S.aureus *by the slide coagulase test (Staph latex kit; Prolex Neston UK) and by the antibiogram using Clinical and Laboratory Standards Institute. Surveillance samples were processed by enrichment and culture. Swabs were placed in nutrient broth (Oxoid, Baskingstoke, UK) supplemented with 10% salt and incubated in ambient air for 18-24 hours. The broth was then subcultured on to Columbia agar (Oxoid) supplemented with 4 mg/L methicillin and incubated for a further 18-24 h in ambient air. All suspect colonies from surveillance swabs were confirmed as MRSA by the slide coagulase test and antibiogram [19].

### Typing of isolates

The first MRSA isolate from each subject was used for in vitro testing and was phenotyped by the Scottish MRSA reference library using PCR and pulsed field gel electrophoresis. The phenotypes of the seven MRSA strains were again confirmed using coagulase, DNAase and methicillin susceptibility tests. The coagulase test was performed according the instruction of ProlexTM Staph Latex Kit (Pro-Lab Diagnostics, UK). For the DNASE test, overnight cultures were spotted onto DNASE agar (Oxoid, UK) and incubated for 37°C for 24 hours. A clear zone around the colony indicated MRSA positive strains. The methicillin susceptibility test was conducted whereby an ME E-test strip (Cambridge Diagnostic Services Ltd. UK) was placed on the surface of the agar freshly swabbed with the test strain. After incubation of the agar at 30°C for 24 hours, the methicillin-induced inhibition zone was recorded.

### In vitro sensitivity

All MRSA bacterial strains and an MSSA reference strain were grown in either nutrient broth (Sigma-Aldrich) or on nutrient agar (1.5% w/v) at 37°C. To determine the antimicrobial activity of acidified nitrite, using HCl as the acid, the assay was performed as described previously [12,20,21]. In brief, the pH of the nutrient broth was adjusted with HCl as this was previously standardized as an assay to create a pH gradient ranging from 1.7 to 7.0 across a micro-titre plate. To achieve a gradient of acidified nitrite, a gradient of potassium nitrite ranging from 0 to 10000 μM was then set up along the length of the micro-titre plate. For all experiments, 8 × 107 cells ml-1 of a stationary phase culture of each strain was used as an inoculum. The micro-titre plate was then incubated at 37*C, 90 rpm and after the designated time, 20 μl aliquots were removed, serially diluted and colony forming units determined by assessing growth on nutrient agar after an overnight incubation at 37°C. The inhibitory effect of acidified nitrite on bacterial growth was determined by measurement of optical density (570 nm) (MRX Microplate Reader, Dynatech) after 24 hours. The minimum inhibition concentration (MIC) for each strain were determined as the average values from 5 replicates. The MIC of acidified nitrite was defined as the lowest nitrite concentration whereby no growth of the strains had taken place at a certain pH after 24 hours.

The protocol was approved by the local Grampian Combined Ethical Committee and Infection Control Committees.

## Results

Patients meeting the entry criteria with MRSA colonisation confined to a wound, with negative swabs elsewhere were rare. Recruiting from an acute hospital with over 1000 beds over 18 months, this limited the study to 8 patients, 6 of whom had more than one colonised wound giving a total of 15 infected wounds. Their clinical details are summarised in table 1. These patients were all treated at the wound sites only with 4.5% citric acid co-administered with 3% sodium nitrite for 5 consecutive days. Swabs were regularly performed from wounds and from other skin sites (see methods). Application of acidified nitrite at these concentrations was well tolerated by all subjects and there was no irritation of the skin noted or increase in pain from the wounds.

**Table 1:** Clinical details and outcomes of wound cultures from subjects treated with acidified nitrite where more than one wound was treated this is shown a several rows.

Age	Sex	Immuno-supressed	Strain tested in vitro	Wounds	Necrosis	Cultures from wound day:				Other sites becoming positive
						0	5	7	9	
84	M	No	3	Gangrene toe	Yes	+	+	+	+	No

90	F	No	4	Amputation	No	+	-	-	-	No

83	F	Prednisolone azathioprine	5	Vasculitic Leg ulcers	Yes	+	+	+	+	No
						+	+	-	-	

85	M	No	6	Orthopaedic pin sites	No	+	-	-	-	No
						+	-	-	-	

74	F	No	7	Pressure sores	No	+	+	+	+	Yes Axilla (day 5)
						+	+	+	+	

74	M	No	8	Orthopaedic pin sites	No	+	-	-	-	No
						+	+	+	+	

79	F	Prednisolone	Not tested	Infected blisters	No	+	-	-	-	No
						+	-	-	-	
						+	-	-	-	

68	F	Renal failure	Not tested	Calciphylaxis ulcers on legs	Yes	+	-	-	+	Yes groins (day 7)
						+	+	+	+	

Following treatment, three patients were completely cleared of their wound infection without recurrence after a further 4 days. Three showed a partial response (clearance of one wound) and two failed to respond. Of the 15 infected wounds 9 (60%) were cleared of MRSA colonisation. Of wounds that responded only 2/9(22%) were necrotic and non-responders were necrotic in 4/6(66%) this difference was not statistically significant (Fisher's exact test).

Although precautions were taken to cover the wounds and use sterile technique when the dressings were changed, re-contamination could occur when dressings were changed or when wetted in the shower. Non-response might also be explained by such recontamination from untreated areas of the patient or close environment. This is supported by the observation that 3 patients developed positive swabs from other previously negative untreated body sites during the study. Four of 7 (57%) of wounds still colonised or recolonised at day 9 occurred in patients with other sites becoming positive while 8 of 8 (100%) of wounds that remained clear occurred in patients not becoming positive at other sites (p = 0.077 Fisher's exact test).

**Figure 1:**
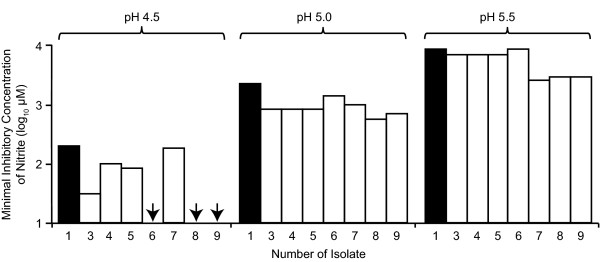
**The effect of pH on the minimum inhibitory concentration of nitrite**. The average minimum inhibitory concentrations (MICs) of nitrite for the MSSA control strain (1), a previously typed MRSA control strain (9) and seven MRSA strains (2-8) isolated from the wounds of patients were determined at either pH 4.5, 5.0 or 5.5, using HCl to acidify. The MICs values shown are an average of five independent determinations for each strain and the arrows indicate that pH 4.5 alone was inhibitory to the strains.

**Figure 2:**
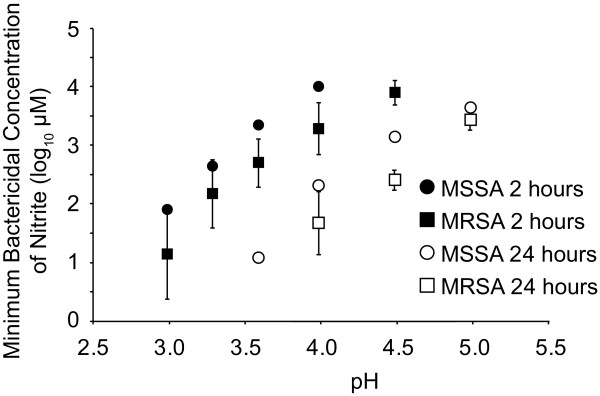
**The effect of pH on the minimum bactericidal concentration of nitrite**. The average minimum bactericidal concentrations (MBC) of nitrite for the seven MRSA strains (2-8) isolated from wounds was compared with the MBC for the MSSA control strain under different pH conditions after either 2 or 24 hours of exposure. HCl was used to acidify the nutrient broth. The error bars represent the standard deviation of the mean MBCs for all seven MRSA strains. At pH 4.5, the maximum concentration of sodium nitrite used in our assay had no effect on the viability of the MSSA strain after 2 hours hence no MBC value is shown.

The first isolate from each patient was used to confirm the phenotype of the MRSA isolate and for *In vitro *testing (Table 2). These showed similar or greater *in vitro *sensitivity to MSSA as measured by MIC at identical pH and nitrite concentrations (Figure 1). As with the MIC analysis, we found that the minimum bactericidal concentration (MBC) of NO_2_^- ^was reduced as the pH of the medium was lowered. In addition, we found that the average MBC to acidified nitrite for the seven MRSA strains was slightly lower than for the MSSA strain, indicating that MRSA strains do not have increased resistance to acidified nitrite. This was particularly evident at pH 4.5, since no killing of the MSSA strain was observed after 2 hours using the maximum concentration of sodium nitrite used in our assay (Figure 2); in contrast, the MRSA strains were still killed under these conditions.

**Table 2:** Typing of isolates.

Strain	Coagulase test	DNASE test	Methicillin susceptibility test*	Reference lab MRSA Phenotype	MRSA Genotype
3	+	+	-	EMRSA 16 variant phage type	PF16d
4	+	+	-	EMRSA 16	PF16a
5	+	+	-	EMRSA 16	PF16a
6	+	+	-	EMRSA 16	PF16a
7	+	+	-	EMRSA 16	PF16a
8	+	+	-	EMRSA 16 variant phage type	PF16d
9 (MRSA) control	+	+	-	**	
1 (MSSA) control	+	+	+	**	

* no inhibition zone up to 256 μg/ml for all MRSA strains

** not done

No adverse effects of the acidified nitrite were reported in this small pilot study.

## Discussion

These pilot results highlight the potential efficacy of acidified nitrite as a topical therapy for MRSA. Considering the lack of an effective therapy in this clinical setting it potentially represents a significant therapeutic advance. Wounds are unlikely to become clear of infection spontaneously. Strains isolated from infected wounds showed *in vitro *sensitivity to acidified nitrite which supports the clinical findings. Indeed, preliminary data suggested that the MRSA strains possibly more sensitive to acidified nitrate than the MSSA strain tested. However, further studies analysing different MSSA strains would be necessary to confirm that this was the case and not due to the individual MSSA strain tested.

Given that there was no difference in the MICs between MRSA isolates from the different subjects, it is unlikely that specific resistance to acidified nitrite could explain failure to clear. Indeed isolate 8 was more sensitive than MSSA *in vitro *and cleared from only one of two wounds suggesting that local factors were responsible. Failure to clear some infected wounds may be due to recontamination from other infected sites as demonstrated in the patient with renal failure. She cleared at one of 2 sites on the legs and the cleared site was re-infected as was a groin swab after 9 days. In all other wounds that cleared, clinical response was maintained to day nine, four days after stopping treatment. Successful treatment was more common in patients who did not have positive swabs at other sites. Finally, wounds that did not clear of MRSA were more frequently associated with tissue necrosis. Topical therapy may not have penetrated a thick eschar. Better bioavailability may result from doubling the concentrations of acid and nitrite which have been used in the treatment of Buruli ulcers [13].

When acid and nitrite are mixed they react together to release nitric oxide (NO) and nitrous oxide (NO_2_). These gases may be irritant to the airways and mucosal surfaces so we avoided treating infections of the nose and limited this study to infections on limited "target" areas of the skin and wounds. As NO is a gas with similar physical properties to oxygen, it can diffuse readily into the skin or a wound to treat the infection and does so more readily than conventional antibiotics.

Previous studies in other micro-organisms [12,21] infer that NO is responsible for the effects seen on organisms. Ghaffari demonstrated that exposure to 200 ppm of NO gas for 5 hours was equally bactericidal to *S. aureus*, MRSA, *Escherichia coli, Group B Streptococcus, Pseudomonas aeruginosa, and Candida albicans *while NO_2 _was not effective [22]. This also suggests that NO is responsible for killing the organism. Ghaffari went on to demonstrate that gaseous NO therapy reduced bacterial counts in experimental staphylococcal skin infections without impairing angiogenesis or wound healing [23]. Miller *et al *exposed cultures of *S. aureus *to eight sub-lethal exposures of NO in order to select for resistant organisms. However, sensitivity was preserved compared to control organisms exposed only to pure air [24]. The precise mechanism whereby NO is bactericidal is not understood but Martinez's recent study examined staphylococci by electron microscopy and observed cellular oedema after 1 hr exposure, increasing destruction of the cell wall architecture after 7 hours, followed by lysis of cells after 24 hours using a nanoparticle technology to deliver NO [25]. Martinez *et al*[25] induced wounds in mice and inoculated these with MRSA. Bacteriological burden measured by culture and by gram staining was significantly reduced with 2 applications of NO nanoparticles after 4 and 7 days respectively [25].

The wide range of infections sensitive to acidified nitrite suggest that this would be a useful addition to the prevention and therapy of MRSA and to wound care, especially where several organisms are responsible. There have been no reports of bacteria becoming completely resistant to acidified nitrite, although nitrite has been used for a very long time as a food preservative so organisms have been exposed to this agent.

## Limitations of this study

As it was difficult to find subjects meeting the entry criteria of infection confined to a wound, numbers of subjects are small. The study also lacked controls or randomisation to prove that the intervention was responsible for the clearing of infection. Finally the concept of subjects having infection confined to a contaminated wound was likely to be erroneous and standardised swabbing protocols do not exclude carriage of MRSA in the patients' intact skin or immediate environment. Infected wounds that did not clear of MRSA did not have resistant infection but were likely to have been recontaminated or alternatively the bioavailability of acidified nitrite was impaired.

## Conclusions

Acidified nitrite is a potential novel therapy for topical application, which can kill MRSA without irritating the skin; this could have important applications in the treatment of localised skin infections, wounds and in the decontamination of hospital staff and merits further study.

## List of abbreviations

MIC: Minimum inhibitory concentration; MBC: Minimum Bactericidal concentration'; MRSA: Methicillin Resistant Staphylococcus aureus; MSSA: Methicillin Sensitive Staphylococcus aureus; NO: Nitric oxide

## Competing interests

ADO and NBB have intellectual property rights for topical acidified nitrite as NO donor therapy for skin infections. They were not responsible for assessing wounds, taking of swabs, or interpretation of cultures and no opportunities for bias were introduced.

## Authors' contributions

ADO conceived the study was senior author responsible for ethics submission, grant and wrote the manuscript. HL Performed all the sensitivity assays on isolates. AAS recruited patients to the clinical study and carried out swabs and clinical assessments. HL performed the *in vitro *sensitivity testing of the organisms. CL provided the laboratory facilities for the study. GF assisted with drafting the manuscript and microbiological expertise. NBB together with ADO was an inventor of acidified nitrite and assisted with concept of the study. All authors read and approved the final manuscript.
